# Hereditary Cancer: Example of a Public Health Approach to Ensure Population Health Benefits of Genetic Medicine

**DOI:** 10.3390/healthcare4010006

**Published:** 2016-01-08

**Authors:** Deborah Cragun, Courtney Lewis, Lucia Camperlengo, Tuya Pal

**Affiliations:** 1 College of Public Health, Department of Global Health, University of South Florida, Tampa, FL 33620, USA; dcragun@health.usf.edu; 2 Moffitt Cancer Center, 12902 Magnolia Drive, Tampa, FL 33612, USA; Courtney.Lewis@moffitt.org (C.L.); Lucia.Camperlengo@moffitt.org (L.C.)

**Keywords:** genetic testing, hereditary breast and ovarian cancer syndrome, genomics, public health

## Abstract

This article introduces the identification, prevention, and treatment of hereditary cancer as an important public health concern. Hereditary cancer research and educational outreach activities are used to illustrate how public health functions can help to achieve health benefits of genetic and genomic medicine. First, we evaluate genetic service delivery through triangulating patient and provider survey results which reveal variability among providers in hereditary cancer knowledge and genetic service provision. Second, we describe efforts we have made to assure competency among healthcare providers and to inform, educate and empower patients with regard to the rapidly evolving field of genomics and hereditary cancer. Lastly, key policy-issues raised by our experiences are discussed in the context of how they may help us to more effectively translate future genomic technologies into practice in order to attain population health benefits from genetic and genomic medicine.

## 1. Introduction

The National Human Genome Research Institute defines genetics as the study of genes and their role in inheritance [1]. As an example, hereditary cancers occur when an individual inherits a mutation in any one of many different cancer predisposing genes that confers high lifetime risks for cancer. Inherited gene mutations cause an individual’s cells to be one step closer to cancer; however, additional gene mutations in several other genes must accumulate during a person’s lifetime in order for cancer to develop. The study of all of a person’s genes as well as interactions between those genes and the environment is referred to as genomics [1].

Genetic and genomic medicine involves using genetic or genomic information as part of an individual’s clinical care (e.g., for diagnostic or therapeutic decision making) [1]. Several genetic and genomic discoveries have already gone “from bench to bedside” and have the clear potential to positively impact disease treatment or prevention among individual patients [2]. Genetic counseling and testing for hereditary cancer predisposition is an example of genetic medicine that has been clinically available for nearly 20 years. In recent years testing many cancer predisposing genes at one time using next generation sequencing has become less expensive than testing for two or three genes using older methods. As more genes are included in a single test, the complexity of genetic medicine increases and moves toward the realm of genomics.

Genomic medicine, however, is typically less focused on the inheritance of single genes that confer high risks for disease. An example of cancer genomics involves sequencing of tens or hundreds of genes in a tumor from a cancer patient to help in selecting cancer treatments. An important distinction here is that the gene mutations identified in most tumors are not the result of inherited mutations, but occur after conception and are typically only present in the tumor cells. Tumor sequencing is already being incorporated into clinical care at many cancer centers and hospitals across the U.S..

The next challenge is going “beyond the bedside” to ensure that genetic and genomic tests are leading to sustained and wide-spread population health benefits while preventing adverse outcomes [3]. We discuss how genetic medicine is becoming a public health priority and describe how we have begun to address the challenge of moving “beyond the bedside” using a public health approach to promote the identification and treatment of hereditary cancer.

## 2. Identifying Hereditary Cancer Risk: A Public Health Priority

Targeted therapies and cancer prevention or screening options may be refined based on inherited gene mutations [4,5]. One of the most widely publicized cases of genetic medicine in the United States (US) is the story of Angelina Jolie who, due to her striking family history of cancer (including several members with breast cancer and mother who died of ovarian cancer), underwent genetic counseling and testing. She was found to have inherited a *BRCA1* mutation that confers increased risks for breast and ovarian cancers of up to 70% and 40%, respectively [6,7,8,9]. To reduce her substantially increased cancer risks, Angelina Jolie elected to have a prophylactic mastectomy in 2013 and prophylactic salpingo-oophorectomy in 2015 [10,11].

As this real-world experience illustrates, identifying hereditary cancer predisposition is of critical public health importance because it changes cancer risk management options and enables patients and their at-risk family members to benefit from proven cancer prevention or early detection options, which can reduce risks to near that of the general population [12,13]. Moreover, identification of hereditary cancer is starting to impact treatments and chemoprevention [4,14].

Evidence supporting the health benefits of using genetic tests and family health history in clinical practice is reflected by the addition of genomics objectives to Healthy People (HP) 2020 [15]. The first genomics objective is to “Increase the proportion of women with a family history of breast and/or ovarian cancer who receive genetic counseling.” The second is a provisional objective, to “Increase the proportion of persons with newly diagnosed colorectal cancer (CRC) who receive genetic testing to identify Lynch syndrome (or familial CRC syndromes)”.

**Figure 1:**
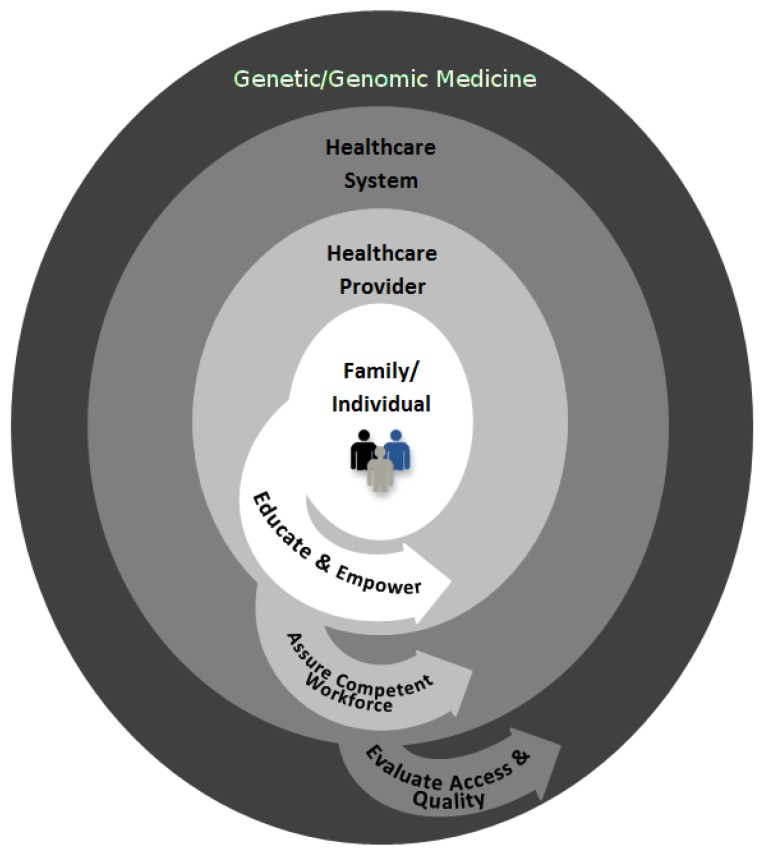
Engagement in public health functions (illustrated by the arrows) at multiple levels can help to achieve the population health benefits of genetic and genomic medicine.

Achieving these HP objectives requires the effective translation of genetics and genomics into healthcare practice through engagement in public health functions at multiple levels, including the patient/family, healthcare providers, and healthcare system [16] (see Figure 1). In the context of public health, it is critical to evaluate the accessibility and quality of genomic and genetic services. Evaluation may identify the need to develop policies to improve access, quality, and/or effectiveness of service delivery. Finally, larger public health efforts are needed to mobilize partnerships in order to assure a competent workforce who will be able to implement genetic and genomic medicine and to educate and empower the patients and families about testing. Examples in the following sections use our experiences with hereditary cancer to illustrate the importance of public health functions in genetic and genomic medicine.

## 3. Evaluating Hereditary Cancer Service Delivery

Ongoing efforts to evaluate genetic service delivery are critical in the context of hereditary cancer given the evolving landscape in which services are provided and the large variety of healthcare providers who offer these services. We have assessed service delivery through surveying Florida healthcare providers who order genetic testing for hereditary breast and ovarian cancer to determine their awareness, knowledge, and practices at two different time points (2010 and fall 2013). Overall, survey results were consistent across both time points in revealing significantly higher knowledge and greater awareness of recent changes in genetic testing and policies among those providers with a professional degree in genetics compared to those with little or no formal training in genetics [17]. Additionally, a high degree of variability was found in genetic service provision across providers. For example, those with a professional genetics degree were more likely than other respondents to report taking a 3-generation family history, spending more time discussing testing with patients, reviewing the possibility that testing could identify an uncertain result, discussing insurance implications of genetic testing, and obtaining written informed consent for testing.

Although we recognize several limitations associated with self-reported data, the results are consistent with our research findings from surveys of patients who had genetic testing. Specifically, when a provider with a master’s degree in genetic counseling or a fellowship-trained MD in clinical genetics was involved, patients were more likely to recall having their family history taken, reviewing the possibility of uncertain results, and discussing insurance-related issues [18]. Triangulating findings from all of our evaluations reveal the need for ongoing educational outreach efforts, particularly given the evolving landscape of genetics/genomics.

## 4. Assure Workforce Competency and Patient Empowerment through Education

**Figure 2:**
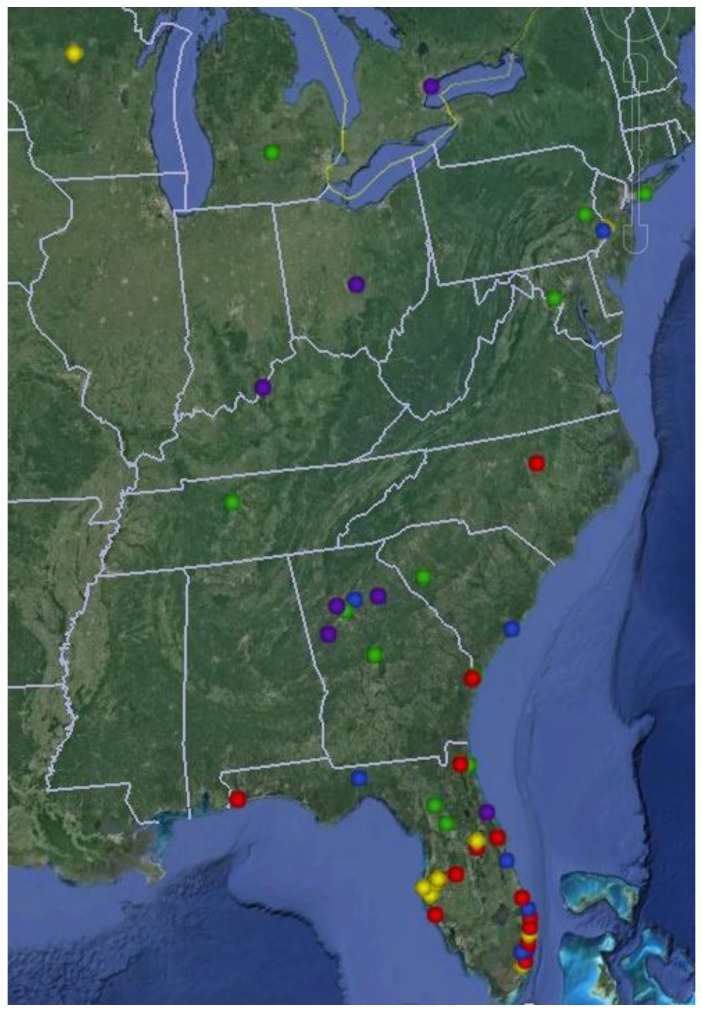
Map showing locations of Inherited Cancer Registry (ICARE) partners.

Recognizing the need for educational outreach to practitioners who provide hereditary cancer services in Florida, we created a network of providers through our Inherited Cancer Registry (ICARE) initiative in 2010. Providers are based at centers across Florida and beyond (Figure 2); and include genetic counselors, nurse practitioners, nurses, physician assistants, physicians and other healthcare professionals who provide genetic testing services [19,20]. Through this infrastructure, providers have access to certified genetic healthcare providers at our center who can help to provide decision support. Since initiation of this effort, we have regularly provided outreach and education to over 180 providers from 141 centers, many of whom attend our bi-monthly web-based case conferences. This network continues to expand as more providers continually request access to education and outreach provided through ICARE which in turn has led to tremendous growth of our bi-monthly virtual case conferences (with providers from 15 to 20 unique sites participating each time).

Through our ICARE initiative, we have also established a research registry of high risk patients, to which over 1700 patients have been consented since 2010, including 945 BRCA carriers. Baseline data is collected on participants through a questionnaire, aimed at addressing our own research questions and contributing to the larger research mission through sharing de-identified data with collaborators to answer various clinical questions and perform genetic association studies. Registry participants are provided with clinical and research updates through a mailed bi-yearly ICARE newsletter (available at: http://inheritedcancer.net/newsletters) [21] and are contacted as new research opportunities become available. Feedback on our newsletters demonstrate how they have positively impacted patients by helping them access appropriate care and keep up to date on evolving practices, particularly about new tests that have become available to identify other genes associated with hereditary cancer and new treatment or screening options. As more genetic and genomic tests are used, the need for ongoing education of both patients and providers is expected to grow. Our ICARE initiative offers one example of a multi-level patient/provider approach that can be used to help address educational needs.

## 5. Public Health Policy-Making in an Era of Genetic/Genomic Medicine

Assuring genetic/genomic competency among healthcare providers will be an increasingly important public health function as testing expands because harms have been documented due to lack of access to appropriate genetic testing, inaccurate results interpretation, or failure to tailor recommendations based on the individual’s personal and family medical history [22,23,24]. The potential for harm, in conjunction with our provider and patient assessments demonstrating lack of knowledge and wide variability in testing practices, raise several policy issues and questions [17,18]. Some of these are discussed below along with approaches that may help ensure genetic and genomic medicine enhances population health and reduces the potential for harm.

### 5.1. How Should Quality in Genetic/Genomic Service Delivery be Defined and by Whom?

Variability we documented in genetic service delivery raises a key question regarding what constitutes quality in genomic services and who should make decisions about quality. Several organizations have developed professional guidelines for elements to include in a pre-test discussion related to hereditary cancer testing [25,26,27,28]. Most of these guidelines recommend providing information about possible test results, yet many providers who responded to our surveys do not discuss the possibility of an uncertain result prior to testing.

As we have begun testing for more genes or even the whole exome (consisting of all protein-coding genes), it is no longer feasible to present highly detailed information about each gene or all possible test results. Subsequently, even providers with formal genetics training are struggling to identify “best practices” for cancer genetic service delivery in this evolving landscape and there remains an unmet need to define quality genetic service delivery based on patient outcomes research rather than relying on anecdotal experience or expert opinions. Empirically determining what information people actually can and will benefit from in their decision making processes in various contexts will be critical for assuring population health benefits as genetic/genomic medicine expands.

### 5.2. Who Should Deliver Genetic/Genomic Services and What Training is Needed?

Genetic and genomic medicine entail performing appropriate risk assessment, genetic/genomic testing, results interpretation, and patient counseling, all of which are competencies included in accredited Master’s level genetic counseling training programs in North America. Although genetic counselors may be well equipped to help integrate genetic/genomic tests into practice, the majority of clinical testing for hereditary cancer within the U.S. is currently ordered in community settings by providers without a professional degree in genetics; and it is anticipated that this will be the case for other genetic and genomic tests in the future.

The Commission on Cancer guideline indicates cancer genetic services may be provided by an informed health care provider with no special certification [28]. However, readily available criteria by which to assess the level to which providers are informed and competent are lacking. Consequently, healthcare providers will need ongoing education and/or assistance from genetic professionals in order to effectively implement genetic medicine.

Policy makers have proposed that health care professionals should, at a minimum, possess the following genomics competencies: (1) a basic understanding of the rationale behind the genomic tests; (2) the ability to identify the limits of his/her genomic expertise; and (3) knowledge regarding where to go for information, resources, and referrals [29,30]. Additional genomics competencies will be necessary for health care professionals who will be ordering and/or interpreting test results in order to ensure that medical recommendations are appropriate.

Evaluating genetic/genomic test results within the context of family history, environmental, and lifestyle factors increases the complexity of prevention interventions and medical care. Recent advances in genetic testing technology have made the option of multi-gene and whole exome testing more financially feasible in clinical practice. Additionally, there are a growing number of genetic testing laboratories offering a variety of hereditary cancer testing options. As such, the increasing complexity of results interpretation that comes with broad-scope testing and the expanding options for hereditary cancer testing highlight the importance of ongoing patient and provider education. Our approach to building a network as part of ICARE is one model by which to discuss challenging cases and educate both providers and patients in the evolving landscape of genetics.

### 5.3. How Can Access to Genetic/Genomic Services Best be Assured?

Amidst issues of quality and competency in genetic service delivery, there are also concerns that restricting genetic services to be delivered only by those with specialized training may further reduce utilization of genetic services [31]. This raises questions about how to train more genetic professionals or provide sufficient genetic/genomics training for other healthcare providers to assure an adequate workforce. To increase the workforce of providers with Master’s training in genetic counseling, the National Society of Genetic Counselors has been trying to address several issues, including: (1) ways to increase the number of students accepted to genetic training programs and/or the creation of more programs; (2) obtaining recognition of master’s trained genetic counselors as healthcare providers under Medicare/Medicaid; and (3) improving reimbursement for genetic counseling services. In addition to these efforts, other coordinated efforts will almost certainly be needed to engage and educate other types of clinicians in order to assure that patients have access to genetic/genomic services that are delivered by competent providers. Knowledge of both cancer genetics (*i.e.*, familial and hereditary cancers) and cancer genomics (specific to tumors) will be needed to advance genetic and genomic medicine in the oncology setting.

## 6. Conclusions

This article provides examples of how we have applied a few of the public health functions to the identification and treatment of hereditary cancer to provide insights that may be useful for ensuring successful implementation of other genetic and genomic medicine applications. Although our examples focused on genetic medicine, many of the challenges and questions related to quality and access are applicable to other genomic medicine applications and public health functions are necessary to ensure that genomic technologies lead to population health benefits for all. As genetic/genomic medicine continues to evolve, ongoing evaluation will be needed to monitor dissemination and implementation in various contexts by various providers. Additionally, ongoing educational interventions are needed to assure healthcare providers are prepared and competent to implement genetic/genomic medicine. In conjunction, consideration should be given with regard to policies that that may be needed to define quality of genetic/genomic medicine, promote wide-spread dissemination of evidence-based practices, and assure a competent work force. Ultimately, it will be important to bring various stakeholders together to develop efficient identification and referral systems to facilitate appropriate utilization of genetic and genomic services.
